# Generation of red blood cells from stem cells: Achievements, opportunities and perspectives for malaria research

**DOI:** 10.3389/fcimb.2022.1039520

**Published:** 2022-11-14

**Authors:** Timothy J. Satchwell

**Affiliations:** School of Biochemistry, University of Bristol, Bristol, United Kingdom

**Keywords:** malaria, red blood cell, reticulocyte, erythropoiesis, stem cell, erythroid, *Plasmodium*

## Abstract

Parasites of the genus *Plasmodium* that cause malaria survive within humans by invasion of, and proliferation within, the most abundant cell type in the body, the red blood cell. As obligate, intracellular parasites, interactions between parasite and host red blood cell components are crucial to multiple aspects of the blood stage malaria parasite lifecycle. The requirement for, and involvement of, an array of red blood cell proteins in parasite invasion and intracellular development is well established. Nevertheless, detailed mechanistic understanding of host cell protein contributions to these processes are hampered by the genetic intractability of the anucleate red blood cell. The advent of stem cell technology and more specifically development of methods that recapitulate *in vitro* the process of red blood cell development known as erythropoiesis has enabled the generation of erythroid cell stages previously inaccessible in large numbers for malaria studies. What is more, the capacity for genetic manipulation of nucleated erythroid precursors that can be differentiated to generate modified red blood cells has opened new horizons for malaria research. This review summarises current methodologies that harness *in vitro* erythroid differentiation of stem cells for generation of cells that are susceptible to malaria parasite invasion; discusses existing and emerging approaches to generate novel red blood cell phenotypes and explores the exciting potential of *in vitro* derived red blood cells for improved understanding the broad role of host red blood cell proteins in malaria pathogenesis.

## Introduction

Infection of humans by parasites of the genus *Plasmodium* that cause malaria results in approximately 240 million clinical cases and 627,000 deaths per year according to recent statistics (WHO, 2021). Most deaths occur in children under the age of five and more than 90% are concentrated in endemic regions of western and Sub-Saharan Africa. *Plasmodium* parasites possess a complex life cycle that includes stages in both the female Anopholes mosquito and the human liver; however, all of the symptoms that characterise this disease occur as a result of the human blood stage in which successive rounds of invasion, intracellular replication within, egress and reinvasion of circulating red blood cells enables the parasites exponential expansion.

Red blood cells, the body’s most abundant cell type and the host for asexual replication of malaria parasites, are highly specialised cells, uniquely adapted for their primary function of delivery of oxygen around the body. They contain no nucleus or intracellular organelles, have a finite lifespan of approximately 120 days, a cytoplasmic protein component dominated by abundant haemoglobin and a unique membrane-cytoskeletal architecture that facilitates deformation and transit through sub-cellular diameter microcapillaries.

**Figure 1:**
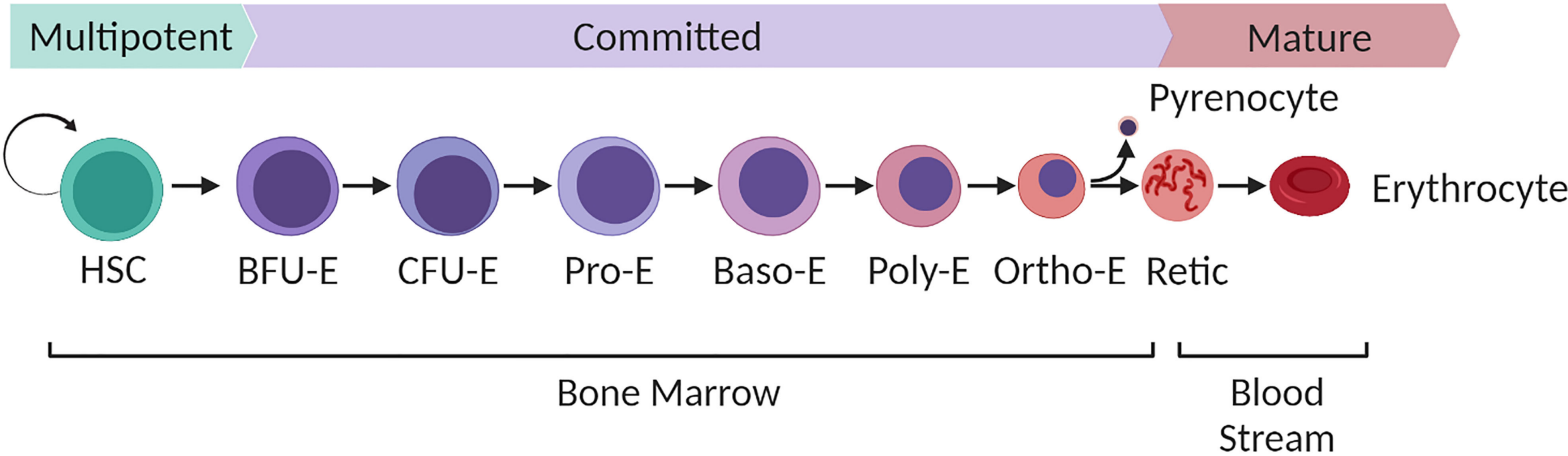
Overview of human bone marrow adult definitive erythropoiesis. Multipotent haematopoietic stem cells with long term self-renewal capacity differentiate along a continuum of cell stages defined by their expansive capacity, degree of commitment to the erythroid lineage and morphological characteristics as illustrated. Burst forming units (BFU-E), the earliest committed erythroid cells expand predominantly in response to stem cell factor with the combined synergistic influence of erythropoietin driving continued proliferation of Colony forming units (CFU-E). Terminal differentiation of resultant proerythroblasts to enucleated reticulocytes takes place within the bone marrow niche within erythroblastic islands that consist of multiple differentiating erythroblasts docked to a central macrophage. Terminal differentiation is characterised by progression through a series of morphologically defined stages (proerythroblast, basophilic erythoblast, polychromatic erythroblasts, orthochromatic erythroblast and reticulocyte). The process is characterised by loss of cellular volume, haemoglobinisation, expression of erythroid specific genes, nuclear condensation, organelle degradation and extrusion of the condensed nucleus (pyrenocyte) to generate a nascent reticulocyte. The pyrenocyte is phagocytosed by the macrophage and the reticulocyte enters the bloodstream, completing its maturation to definitive biconcave erythrocyte in the circulation over the subsequent 24-48 hours. Figure created with BioRender.com.

In humans red blood cells are generated through a specific process of stem cell differentiation known as erythropoiesis. Proerythroblasts, first derived from multipotent haematopoietic stem cells, undergo a complex process of differentiation that occurs in contact with macrophages in so-called erythroblastic islands within the bone marrow niche. Driven by the actions of the glycoprotein hormone erythropoietin, erythroblasts undergo a series of cell divisions in which the cell dramatically transforms both transcriptionally and morphologically: reducing its volume, haemoglobinising, remodelling its membrane properties, degrading intracellular organelles whilst condensing and ultimately expelling its own nucleus, which is phagocytosed by the erythroblastic island macrophage to leave a nascent anucleate reticulocyte (**Figure 1**). The reticulocyte exits the bone marrow, entering the circulation where it completes its maturation to generate the characteristically familiar biconcave erythrocyte. In healthy adults, the continuous loss or clearance of the finitely life-spanned red blood cell is maintained in equilibrium through the ongoing production of new red blood cells at a rate of around 2 million cells every second (Palis, 2014).

Efforts to recapitulate the process of erythropoiesis *ex vivo* with the ultimate goal of producing *in vitro* derived red blood cells as a transfusion product have been longstanding and varied. Myriad approaches that address the key obstacles of efficient terminal differentiation and enucleation, sustainability, yield, scalability and cost have been and continue to be developed to this end (Lim et al., 2021; Pellegrin et al., 2021). For the malaria research community, enabled access *ex vivo* to invasion susceptible erythroid cells of increasing interest such as reticulocytes and erythroblasts in quantities amenable to study offers new opportunities for insight. Perhaps most excitingly however the capacity for derivation of red blood cells from their nucleated precursors *ex vivo* has opened the door to genetic manipulation of red blood cells and the opportunity for targeted exploration of the role of host red blood cell proteins in multiple aspects of malaria pathogenesis.

This review will highlight applications of erythroid stem cell biology to the study of malaria conducted to date, discuss alternative approaches for the generation of *in vitro* derived erythroid and red blood cells, their respective advantages and disadvantages and highlight challenges and opportunities in the application and use of such cells for malaria studies.

## *In vitro* erythropoiesis – Primary cell models

Although multiple sources of erythroid precursors have since been demonstrated, with different degrees of efficiency and success, to undergo terminal differentiation, the most widely applied approach thus far utilises primary haematopoietic stem cells isolated from bone marrow, umbilical cord or peripheral blood. Expansion of a subset of stem cells, enriched initially through CD34+ cell isolation (Giarratana et al., 2005; Giarratana et al., 2011; Griffiths et al., 2012) or expanded directly from peripheral blood mononuclear cells (Van Den Akker et al., 2010) using a cocktail of growth factors that include erythropoietin, interleukin 3 and stem cell factor allows for the expansion of large numbers of proerythroblasts that can be differentiated to generate enucleated reticulocytes (young red blood cells). Reticulocytes generated using this approach exhibit similar characteristics to *in vivo* derived reticulocytes (Giarratana et al., 2011; Griffiths et al., 2012; Moura et al., 2018; Heshusius et al., 2019), undergo maturation to biconcave erythrocytes upon transfusion (Giarratana et al., 2011; Kupzig et al., 2017) and showed favourable circulatory half-life in a proof of principle human clinical trial (Giarratana et al., 2011).

Crucially, in the context of malaria, reticulocytes derived through *in vitro* culture of primary HSCs have been demonstrated in multiple studies to support invasion by malaria parasites *Plasmodium falciparum* (Tamez et al., 2009; Bei et al., 2010; Fernandez-Becerra et al., 2013; Egan et al., 2015) and *plasmodium vivax* (Panichakul et al., 2007; Noulin et al., 2012; Roobsoong et al., 2015; Kanjee et al., 2021) with several of these studies also reporting successful invasion of late stage nucleated orthochromatic erythroblasts.

## Sustainable sources of erythroid cells

Whilst there are many advantages to the use of primary HSCs, the finite proliferative capacity of this cell source, challenges of efficient genetic manipulation and need for repeated transductions between experimental cultures each from a new donor source have encouraged the search for more sustainable sources of erythroblasts.

### Induced pluripotent stem cells

The development of methodology allowing for the reprogramming of somatic cells by expression of four transcription factors (Oct4, Sox2, c-Myc and Klf4) to pluripotency was a landmark in stem cell biology (Takahashi et al., 2007; Yu et al., 2007). The initial promise that accompanied the development of induced pluripotent stem cells (iPSC) that could be directed into the erythroid lineage as a limitless robust source of *in vitro* derived red blood cells however has yet to be fulfilled. Conceptually iPSC cell lines offer a level of sustainability, versatility and genetic tractability that makes them a valuable alternative to primary HSCs. However, difficulties associated with variation between lines, persistence of embryonic and/or fetal haemoglobin, low rates of expansion, incomplete differentiation and poor enucleation as well as the complexity of protocols required to differentiate such cells have plagued the quest for iPSC generated red blood cells (Dias et al., 2011; Trakarnsanga et al., 2014; Focosi and Pistello, 2016). Efforts to improve methodologies for the derivation of such lines and their subsequent differentiation remain a highly active area of research (Bernecker et al., 2019; Hansen et al., 2019; Lim et al., 2021), however application of such lines for generation of cells suitable for malaria studies so far is extremely limited (Pance et al., 2021).

### Immortalised erythroid cell lines

Generation of immortalised erythroblast cell lines, capable of infinite proliferation whilst retaining the capacity to undergo terminal erythroid differentiation and enucleation has presented a holy grail within the erythroid biology research community. Such lines could provide a sustainable source of isogenic erythroid precursors, be readily genetically manipulated, selected or clonally screened for the derivation of modified sublines and cryopreserved for long term and repeated experimentation. Immortalisation at a committed stage of erythropoiesis also reduces the culture time required to obtain reticulocytes, which for culture from HSCs takes approximately 18-21 days.

Retention of capacity for complete terminal differentiation whilst maintaining a state of continuous proliferation is a significant biological and technological difficulty. Long established erythroleukemic cell lines such as K562 (Lozzio and Lozzio, 1975) and HEL cells do not faithfully recapitulate aspects of normal erythropoiesis including the key step of enucleation (Kanjee et al., 2017) and thus are not capable of generating cells suitable for malaria studies. Some such lines are receptive of chemical induction to haemoglobinise and/or undertake stunted differentiation. The JK-1 cell line for example can be induced to generate nucleated erythroblasts with a polychromatic erythroblast-like morphology that notably support invasion by *P. falciparum* (Kanjee et al., 2017). Readily genetically manipulatable, these cells provide a means of insight into requirement of host receptors for successful attachment and invasion and were employed to investigate a functional association between host receptors basigin and CD44 during *P. falciparum* invasion. Nevertheless, in interpreting effects or excluding contribution of proteins using this nucleated cell model it is important to appreciate that cytoplasmic and membrane composition as well as context and presentation of proteins differs between cells pre and post enucleation and care must be taken in extrapolating mechanisms to circulating red blood cells. JK-1 cells do not support further parasite development beyond initial invasion excluding their use to study other aspects of parasite development and pathology.

In 2017, Trakarnsanga et al. reported the first adult human immortalised erythroblast cell line capable of undergoing terminal erythroid differentiation and enucleation to generate functional adult reticulocytes that express beta globin (Trakarnsanga et al., 2017). Unlike erythroleukemic cell lines, BEL-A cells were immortalised by expression of a doxycycline inducible HPV16 E6/E7 construct in healthy adult bone marrow CD34+ HSCs using an approach first employed by Kurita et al. for the generation of HIDEP (iPSC-derived) and HUDEP (cord blood derived) erythroid progenitor cell lines (Kurita et al., 2013).

BEL-A cells can be maintained in continuous expansive culture through supplementation with erythropoietin, stem cell factor, dexamethasone and doxycycline and can be induced to undergo differentiation by transition to a differentiation media that includes erythropoietin, human serum and holotransferrin and through the removal of doxycycline yielding a mixed culture comprising apoptosed cells, orthochromatic erythroblasts and enucleated reticulocytes (Trakarnsanga et al., 2017; Hawksworth et al., 2018). Reticulocytes derived through BEL-A cell differentiation can be purified by leukofiltration and are proteomically equivalent to reticulocytes derived from primary HSCs (Trakarnsanga et al., 2017).

Whilst an obsession with ‘enucleation percentages’ that do not incorporate variations in cell expansion during differentiation, viability and lineage purity between different systems and cell lines is often unhelpful (Daniels et al., 2020), current literature suggests this adult bone marrow derived erythroblast line to give the greatest reticulocyte yield amongst similar equivalents (Kurita et al., 2013; Kurita et al., 2019; Scully et al., 2019; Daniels et al., 2020).

Unquestionably rates of conversion of orthochromatic erythroblasts derived from cell lines to reticulocytes at present fail to match those observed in primary HSC derived cells. This is perhaps unsurprising given the tight regulation of cell cycle involved in both cell replication and enucleation (Daniels et al., 2021; Wang et al., 2022) and the requirement for its dysregulation in order to facilitate erythroblast immortalisation (Daniels et al., 2021). However, despite this, the sustainable nature of this erythroblast source and the opportunity for sophisticated genetic manipulation that it enables allows several advantages as an alternative for generation of novel host cell models as discussed below.

## A route to the inaccessible

### New insight into the role of erythroblast infection

The capacity for malaria parasites to infect cells within the erythroid lineage that are not definitive circulating erythrocytes has long been recognised (Marchiafava and Bigmani, 1894; Craik, 1920; Shushan and Adams, 1937). Increased tropism for (in the case of *P. falciparum* (Wilson et al., 1977; Pasvol et al., 1980)) and restricted infection of (in the case of *P. vivax* (Craik, 1920; Hegner, 1938)) reticulocytes has been accepted for decades. Observations of parasitised erythroblasts in bone marrow was reported as early as 1894 (Marchiafava and Bigmani, 1894; Feldman and Egan, 2022). Only more recently however have questions been raised as to the possible reasons for and implications behind infection of more immature erythroid cells. One reason for this is the difficulties associated with obtaining immature erythroid cells in quantities amenable to controlled invasion studies *ex vivo*.

*In vitro* culture of erythroid cells has allowed for the generation of proerythroblasts and subsequent intermediately differentiated erythroblasts that can be exposed to malaria parasites for tracking of both host and parasite cellular development. In 2009 Tamez and colleagues used *in vitro* derived erythroid cells to assess stage specific susceptibility of human erythroblasts to *P. falciparum* infection, reporting efficient invasion and intracellular development of parasites within orthochromatic erythroblasts (Tamez et al., 2009).

More recently, Neveu et al. employed the same approach to investigate the reported presence of sexual stage gametocytes in bone marrow and the prospective role of erythroblast infection as an enabler of gametocytogenesis (Neveu et al., 2020). In their study, Neveu and colleagues demonstrate that nucleated polychromatic erythroblasts support immature (sexual stage) gametocyte development from stage I to IV for 8 days leading to the production of mature gametocytes within reticulocytes as both parasite and host cell continue to differentiate. Gametocyte development was observed to slow the differentiation of the host erythroid cell, increasing the period in which the cell remains nucleated to complete its development prior to enucleation and the release of mature gametocyte containing reticulocytes into the circulation for subsequent transmission.

**Figure 2:**
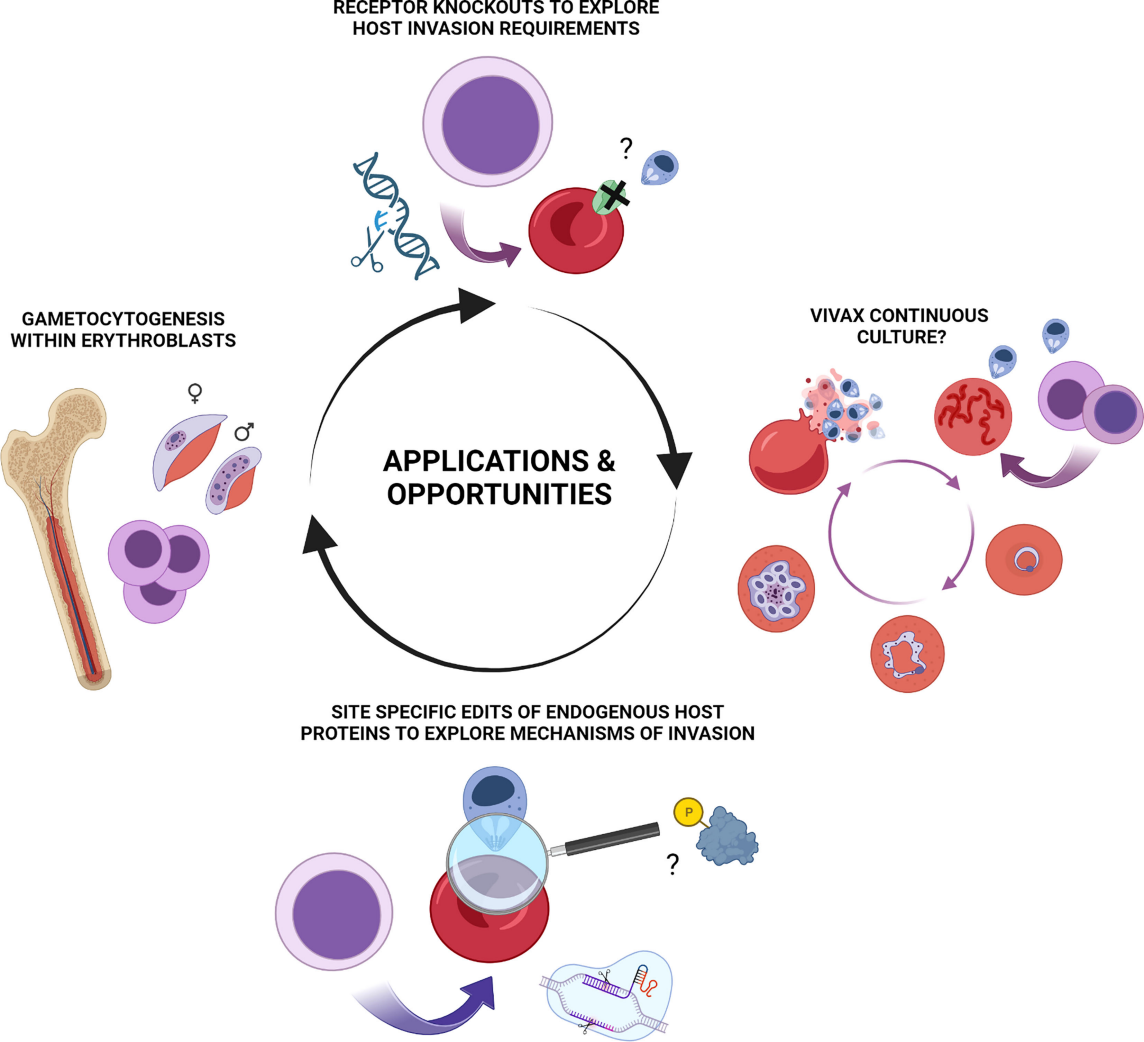
Summary of applications and potential opportunities for erythroid stem cell biology to study of malaria pathogenesis. Illustrative summary of applications and opportunities which include generation of novel *in vitro* derived red blood cell phenotypes for exploration of host protein contribution to invasion, host cell remodelling and other aspects of pathogenesis of blood stage *Plasmodium falciparum* and Plasmodium vivax infection. CRISPR-mediated NHEJ mediated knock outs, lentiviral rescue experiments and HDR mediated site-specific editing of endogenous loci are each applicable to *in vitro* erythroid cultures. Emerging and future opportunities include exploration of nucleated erythroblasts as a preferential host environment for gametocytogenesis and the long-standing enigma and quest for a means of continuous *ex vivo* propagation of *Plasmodium vivax.* Figure created with BioRender.com.

Gametocytogenesis is known to involve extensive remodelling of host cell properties and co-option of host protein components (Tiburcio et al., 2012; Tiburcio et al., 2015; Neveu and Lavazec, 2019). The nucleated erythroid cells in which this newly identified aspect of gametocytogenesis takes place however exist only within the experimentally inaccessible bone marrow in humans. The demonstration that *in vitro* derived erythroblasts recapitulate this intriguing process thus provides a window to study this complex aspect of malaria pathogenesis by application of stem cell biology to malaria as well as a potential *ex vivo* model for putative drug screening approaches. Exploitation of new tools that allow for genetic manipulation of these host cells in addition to cellular resources and lines generated in other areas offers much potential for future insight in this area (**Figure 2**).

### The much-sought reticulocyte

The widely accepted endpoint of most successful *in vitro* erythroid culture systems is the youngest class of circulating red blood cells, otherwise known as reticulocytes (Giarratana et al., 2011; Griffiths et al., 2012; Shah et al., 2014; Heshusius et al., 2019; Pellegrin et al., 2021; Bernecker et al., 2022). The immediate precursor to the mature biconcave erythrocyte, the term reticulocyte describes enucleated cells (of evolving maturity) that are generated following extrusion of the erythroblast condensed nucleus (pyrenocyte) and *in vivo* exit the bone marrow to enter the bloodstream and circulate the body, remodelling their membrane to achieve biconcavity as they do so. Although able to effectively function in the same way as the slightly more mature definitive erythrocyte, reticulocytes are larger in size, contain residual RNA (classically detectable with nucleic acid binding dyes such as thiazole orange) and retain expression of the transferrin receptor CD71 (at varying levels) that is lost progressively during maturation (Malleret et al., 2013; Ovchynnikova et al., 2018; Stevens-Hernandez and Bruce, 2022).

Reticulocytes can be successfully invaded by a broad range of malaria parasites including *P. falciparum*, *knowlesi*, *ovale* and *vivax* (Mcqueen and Mckenzie, 2004). *P. falciparum*, which is responsible for the most severe form of malaria exhibits a variably reported preference for reticulocytes (Wilson et al., 1977; Pasvol et al., 1980; Lim et al., 2013), this may reflect the increased surface area for attachment, differences in membrane tension or energetic state of these newly generated cells. *P. vivax* invasion in contrast is restricted to these more immature red blood cells, which account for just 0.5-1% of circulating red blood cells. Since reticulocytes can be invaded by multiple species of *Plasmodium* (even where essential host surface receptors required differs), they provide an extremely valuable model to generate insight into surface receptor independent, or downstream aspects of malaria parasite invasion and host protein co-option that may be conserved across species.

For the study of *P. vivax* invasion, access to reticulocytes in sufficient quantity and of sufficient levels of purity to be useful for invasion studies has proved a major obstacle for many years. The capacity for *in vitro* stem cell differentiation to derive relatively large, pure populations of these cells from a single donor without the need to pool samples (e.g. from multiple cord blood units) has already recently yielded insight into variation in receptor reliance that exists between strains (Kanjee et al., 2021).

Famously, the study of *P. vivax* has been hampered by the absence of the kind of *in vitro* system for continuous maintenance of parasites in culture with which Trager and Jensen revolutionised *P. falciparum* research (Trager and Jensen, 1976). The recent demonstration of sustained *P. vivax* blood stage infection and transmission in a humanised mouse model with human HSPC transplantation is an exciting development (Luiza-Batista et al., 2022b). The observation that much of the parasite biomass (comprising asexual and in particular sexual parasite stages) occurred in the bone and thus the prospective importance of nucleated erythroblast infection adds further intrigue and potential for *in vivo* insights. Similarities between *P. knowlesi* and *vivax* have also been exploited as a means for screening *P. vivax* blood stage malaria candidates (Ndegwa et al., 2021). Despite these advances, the drive for a continuous culture system to propagate *P. vivax* remains. The complexities and obstacles to establishment of such a system are manifold, likely extending beyond solely the requirement for large numbers of permissive reticulocytes (elegantly reviewed elsewhere (Bermudez et al., 2018; Gunalan et al., 2020; Thomson-Luque and Bautista, 2021)). Success in continuous propagation of *P. vivax ex vivo* in reticulocytes of any source has been minimal. Clearly however, in the ability to generate reticulocytes (and earlier erythroid cells) that are susceptible, at least to invasion, by *P. vivax*, stem cell biology has an important role to play whether directly or indirectly in any future development of such a system (**Figure 2**).

## Manipulating host protein expression

Historically, studies of malaria parasite invasion of the red blood cell have focused predominantly upon identification of the proteins on the surface of the host red blood cell (Salinas and Tolia, 2016; Satchwell, 2016) and more fervently, the merozoite itself, that mediate attachment, potentially providing targets for vaccine design (Beeson et al., 2016). Elegant use of proteases, blocking antibodies and the identification and study of rare naturally occurring red blood cell phenotypes with receptor mutations or null phenotypes have provided valuable information regarding the requirements for and functional redundancy of individual receptors and recent years have seen the reporting of a new swathe of receptors with implied or demonstrated roles in merozoite attachment and invasion (Tham et al., 2010; Crosnier et al., 2011; Bhalla et al., 2015; Egan et al., 2015; Egan et al., 2018; Olivieri et al., 2021). However, there remains much that we do not understand regarding the contribution of host proteins both at and beneath the surface of the red blood cell to this process.

Perhaps the biggest obstacle to elucidation of the function and contribution of red blood cell proteins in malaria infection is the inability to manipulate protein expression in the genetically intractable anucleate erythrocyte. Previous reliance upon the identification of often vanishingly rare naturally occurring phenotypes to provide insight is inefficient and precludes hypothesis driven investigation of host protein involvement in invasion. The capacity to derive reticulocytes (young red blood cells) that are susceptible to invasion by malaria parasites through *in vitro* culture and differentiation of haematopoietic stem cells (Tamez et al., 2009; Bei et al., 2010; Fernandez-Becerra et al., 2013; Noulin et al., 2014; Egan et al., 2015) unlocks new possibilities previously inaccessible to red blood cell biologists.

*Ex vivo* access to nucleated erythroid progenitor cells (from various sources as outlined in this review) allows for the genetic manipulation of cells, inducing alterations to protein expression that can be retained during subsequent terminal erythroid differentiation to produce enucleated red blood cells with novel phenotypes. As improvements in erythroid culture methodology and advances in genetic manipulation and gene editing approaches have boomed over the last decade, so the level of sophistication of this approach has, and continues to increase.

### Primary cell successes so far

In 2010 Bei et al. exploited lentiviral transduction of primary CD34+ HSCs to express shRNA for specific depletion of the host EBA175 binding receptor Glycophorin A (Bei et al., 2010). By differentiating these transduced cells, the authors were able to derive reticulocytes with an 80% reduction in expression of GPA that exhibited substantially reduced invasion compared to control by *P. falciparum*, confirming the important role played by this protein and validating the approach for host focused studies of invasion receptor requirements. The same approach was used as part of the seminal identification of basigin as the essential PfRh5 binding receptor necessary for successful *P. falciparum* invasion (Crosnier et al., 2011), by Niang and colleagues to explore GPC-STEVOR binding and rosetting (Niang et al., 2014) and was further expanded by Egan et al. who employed an shRNA library screen to identify roles for CD55 and CD44 in invasion (Egan et al., 2015).

Derivation of *in vitro* cultured reticulocytes from CD34^+^ cells in combination with shRNA provides a powerful system for the identification of candidate receptors important in invasion (Egan et al., 2015). However, the finite proliferative capacity of primary haematopoietic stem cells, necessitating repeat transduction between experiments and limiting selection time, together with the incomplete depletion of receptor expression by shRNA, imposes limitations to the complexity of experiments that can be performed using this approach. Development of sustainable enucleation competent immortalised sources of erythroid cells accompanied by the explosion in CRISPR-Cas9 mediated gene editing advancements have gone a long way toward overcoming some of these issues, though challenges do remain.

### Modified cell lines

One of the major benefits of cell lines over primary cells is the relative ease with which such cells can be manipulated at the genetic level. Expanding BEL-A cells can be lentivirally transduced with high efficiency (Trakarnsanga et al., 2017), are amenable to CRISPR-Cas9 mediated gene editing for the generation of stable clonal cell lines [evidenced through the generation of reticulocytes with knock out of individual and multiple blood groups (Hawksworth et al., 2018; Satchwell et al., 2019)]. Whilst the karyotypic abnormalities and possibility of genetic drift intrinsic to immortalised cell lines is a consideration in gene editing of such cells, the fact that both edited and unedited sublines derive from the same donor eliminating the impact of donor variability and polymorphisms between experiments is an additional advantage. Leukofiltered purified BEL-A derived reticulocytes were demonstrated to support both invasion by and complete intracellular development and egress of *P. falciparum* at rates equivalent to that of primary CD34+ HSC derived reticulocytes (Satchwell et al., 2019). CRISPR mediated knockout of basigin and lentiviral complementation studies were further employed by Satchwell et al. (Satchwell et al., 2019), validating this cell system as a means for interrogation of host protein requirements for successful *P. falciparum* invasion and excluding a requirement for the basigin cytoplasmic domain in invasion. Orthochromatic erythroblasts derived from a similarly immortalised line (EJ) were found to support invasion by both *P. falciparum* and *P. vivax*, albeit less permissively than primary erythrocytes and reticulocytes respectively (likely a reflection of differences in host membrane-cytoskeletal protein context and membrane properties of these more immature cells). DARC (Duffy) knockout and re-expression abrogated and rescued invasion by *P. vivax* respectively (Scully et al., 2019).

## Future opportunities

To date, most insight derived through genetic manipulation of *in vitro* derived red blood cells has come through depletion of expression of host cell surface receptors, first by shRNA (Bei et al., 2010; Egan et al., 2015) and more recently through CRISPR-Cas9 non homologous end joining (NHEJ) mediated gene knockout (Kanjee et al., 2017; Satchwell et al., 2019; Scully et al., 2019; Shakya et al., 2021). In facilitating the knock-down or knockout of receptors for which naturally occurring null phenotypes do not exist or are extremely rare this approach has tremendous value. For studies of *P. vivax*, the ability to generate invasion susceptible reticulocytes (and erythroblasts) and to knock out candidate receptors should increase our understanding of the repertoire of host cell proteins that may be involved in invasion by this species.

As technology and methodologies advance however, so too do the possibilities for further insight using stem cell derived red blood cells. By complementing a BSG knockout (KO) line with a wild type and truncated BSG open reading frame Satchwell and colleagues were able to expand upon the use of KOs alone to interrogate the requirement for a specific intracellular receptor domain for the first time (Satchwell et al., 2019). Expansion of this knock out and mutant rescue approach for dissection of the requirements of different host protein components required for successful invasion will be of future interest. Further, active development of successful protocols for homology directed repair based ‘knock ins’, enabled by the ability to screen immortalised erythroblast clones for site specific edits is an exciting new frontier that paves the way for a more refined dissection of the role played by red blood cell proteins in *Plasmodium* invasion and development.

Site specific requirements of host cell proteins in malaria parasite invasion, development and host remodelling have been widely documented and postulated, with many as yet unidentified contributions undoubtedly still to be uncovered. These range from single nucleotide polymorphisms, sites of glycosylation (Goerdeler et al., 2021) and palmitoylation (Kumari et al., 2022) of receptors to phosphorylation sites within membrane and cytoskeletal adaptor proteins. By enabling their alteration (deletion, disruption or replacement with phospho-modification incompetent residues for example), immortalised erythroid cell lines allow for the role of such sites to be dissected within a true cellular context, providing a powerful tool for new mechanistic insight. Interrogation of the role of specific domains, regulatory sites and residues on endogenously expressed host membrane and cytoskeletal proteins (including exploration of the role of the swathe of reported post-translational modifications associated with invasion (Bouyer et al., 2016; Zuccala et al., 2016; Aniweh et al., 2017; Sisquella et al., 2017) and their relevance to the induction of transient host cytoskeletal clearance (Zuccala and Baum, 2011)) can inform our understanding of malaria pathogenesis and contribute to the increased acceptance of and search for potential host directed therapeutic opportunities (Adderley et al., 2020; Chien et al., 2021; Wei et al., 2021) (**Figure 2**).

## Challenges

For all the exciting opportunities for novel insight use of *in vitro* derived red blood cells present for malaria research, their application to such studies is not without its practical challenges. Manipulating erythroblasts with a view to generation of enucleated red blood cells must consider the need to ensure that the programme of differentiation and enucleation is not compromised, and pleiotropic effects of alterations carefully assessed and considered.

Where donor red blood cells are plentiful and easily accessible, derivation of *in vitro* derived red blood cells, particularly those that have undergone genetic manipulation represents a considerable investment of labour and resource. It is not always possible (or rather, feasible) to replicate assays that may be considered routine using donor red blood cells. Where flow cytometry assays of parasitemia using nucleic acid staining dyes is routine for donor erythrocytes, the confounding nuclear signal where orthochromatic erythroblasts are studied prohibits this means of assessment and in the ideal situation where purified reticulocytes are studied residual RNA in reticulocytes necessitates careful controls (Satchwell et al., 2019). Miniaturisation of assays and manual inspection of cytospin preparations (Bei et al., 2010; Egan et al., 2015; Satchwell et al., 2019; Kanjee et al., 2021) have been powerful enablers of *in vitro* red blood cell use however efforts to adapt and improve flow cytometry based assessment through robust, nucleic acid labelling, use of fluorescent parasite lines (Neveu et al., 2020), label free assessment of parasitemia (Frita et al., 2011; Pance et al., 2021) or *via* advanced technologies such as Imaging Flow Cytometry (Luiza-Batista et al., 2022a) represent important endeavours. Ongoing efforts within the community of researchers seeking to improve and optimise *in vitro* erythroid culture enucleation rates, scalability and purification methods are of continued importance to improve accessibility (Lim et al., 2021; Pellegrin et al., 2021; Gallego-Murillo et al., 2022).

## Conclusions

Malaria is a complex and multi-faceted disease, caused as it is, by a parasite that transitions between multiple forms, residing in multiple host cells throughout its lifecycle, each presenting their own unique difficulties for study. In the case of the red blood cell host, genetic intractability of the mature cell and inaccessibility of its increasingly interesting precursors have presented major historical obstacles to detailed understanding of the ways in which host proteins resist, contribute or are co-opted during malaria invasion and pathogenesis. Stem cell biology and the more recent use of immortalised erythroid cells has already demonstrated its value to malaria research, identifying new and overlooked host protein requirements for invasion and opening up new areas for investigation. We look forward to the exciting new avenues, insights and opportunities for interventions that it may uncover in the coming years.

## Data availability statement

This study did not involve any underlying data.

## Author contributions

TJS conceptualised and wrote the review. The author confirms being the sole contributor of this work and has approved it for publication.

## Funding

TJS was supported by the UK Medical Research Council Grant ID. MR/V010506/1.

## Conflict of interest

The author declares that the research was conducted in the absence of any commercial or financial relationships that could be construed as a potential conflict of interest.

## Publisher’s note

All claims expressed in this article are solely those of the authors and do not necessarily represent those of their affiliated organizations, or those of the publisher, the editors and the reviewers. Any product that may be evaluated in this article, or claim that may be made by its manufacturer, is not guaranteed or endorsed by the publisher.
